# Advances and prospects of 3D air-liquid interface cultured middle ear epithelial cells and inner ear organoids in otologic diseases

**DOI:** 10.3389/fbioe.2026.1881504

**Published:** 2026-09-09

**Authors:** Le Gu, Mengxian Du, Kai Zhang, Yuancheng Wu, Tihua Zheng, Qingyin Zheng, Bo Li

**Affiliations:** 1 Shandong Key Lab of Complex Medical Intelligence and Aging, School of Special Education and Rehabilitation, Shandong Medical and Pharmaceutical University, Yantai, Shandong, China; 2 Department of Rehabilitation Medicine, Yantaishan Hospital, Shandong Medical and Pharmaceutical University, Yantai, Shandong, China

**Keywords:** air-liquid interface, cochlear organoids, hearing loss, middle ear epithelial cell model, personalized pathological model, vestibular organoids

## Abstract

Hearing loss and vestibular disorders represent major otologic disease challenges. Traditional disease models struggle to accurately mimic human otologic pathologies due to species differences and limited physiological relevance. Recent advancements in otic vitro models have provided crucial tools for related research. The core representatives include the middle ear epithelial cell model cultured at the air-liquid interface (ALI) and inner ear organoids. ALI culture of middle ear epithelial cells highly mimics middle ear tissue structure and function, finding applications in drug screening and toxicity assessment. Inner ear organoids facilitate the *in vitro* generation of functional three-dimensional structures containing hair cells, supporting cells, and neurons, while recapitulating both inner ear development and specific disease phenotypes. This review highlights the origins, research progress, and application prospects of these two model types, aiming to provide references for elucidating otologic disease mechanisms, personalized drug screening, and evaluating gene therapy strategies.

## 1 Introduction

**FIGURE 1:**
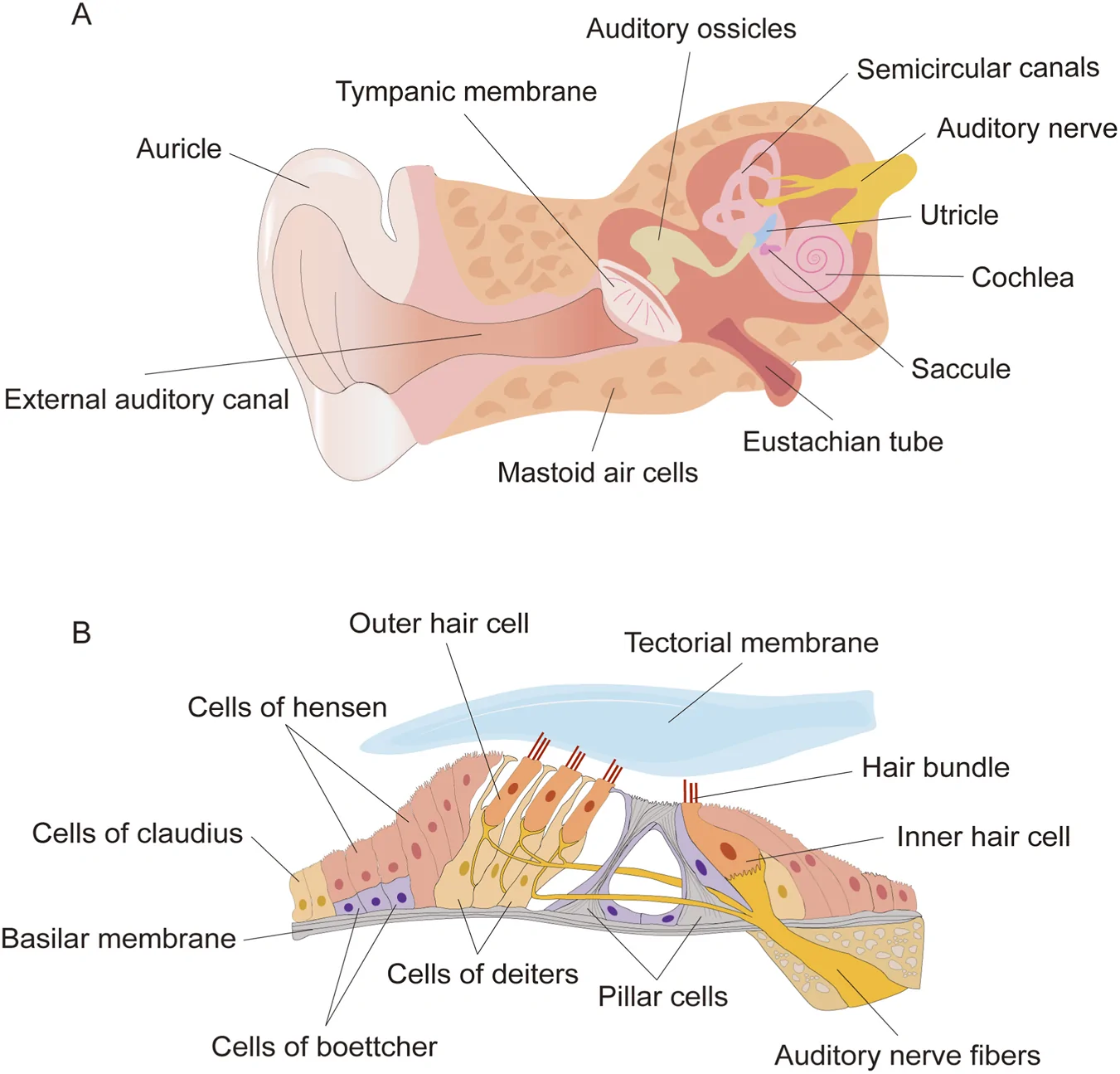
Anatomical structures of the human ear and cochlear sensory epithelium. This figure systematically presents the key anatomical structures involved in sound transmission and auditory signal generation. **(A)** Schematic of the human ear, showing external, middle and inner ear structures including the external auditory canal, tympanic membrane, auditory ossicles, eustachian tube, vestibular apparatus and cochlea. **(B)** Microstructural organization of the cochlear organ of Corti. This panel details hair cells, supporting cells, basilar membrane, tectorial membrane and auditory nerve fibers.

The ear serves as both a biological sensor and a signal processor in the human body. Anatomically, it comprises three distinct regions: the outer ear, middle ear, and inner ear. The inner ear houses the cochlea, responsible for auditory perception, and the vestibular system, which regulates bodily balance. The vestibular system consists of three semicircular canals, the utricle, and the saccule (Anthwal and Thompson, 2016) (Figure 1A). Physiologically, the ear converts acoustic mechanical waves into neural signals through three sequential processes: sound capture by the outer ear, sound conduction by the middle ear, and sound transduction by the inner ear. Together, these processes give rise to auditory perception. Beyond auditory function, it also mediates sound source localization, auditory scene analysis, and maintenance of bodily balance (Ashmore et al., 2010; Middlebrooks, 2015; Cullen, 2023).

According to the World Health Organization, over 1.5 billion people worldwide suffer from varying degrees of hearing loss, with projections indicating this number could reach 2.5 billion by 2050 (Chadha et al., 2021; Prasad et al., 2024). Current clinical treatments exhibit significant limitations, with efficacy highly dependent on the type and cause of hearing loss.

There are three types of hearing loss: conductive, sensorineural, and mixed. Conductive hearing loss occurs when sound is not sent easily through the outer ear canal to the eardrum and the tiny bones (ossicles) of the middle ear. Conductive hearing loss makes sounds softer. This type of hearing loss can often be corrected medically or surgically. Sensorineural hearing loss (SNHL) happens when there is damage to the inner ear (cochlea) or to the nerve pathways from the inner ear to the brain. Most of the time, SNHL cannot be medically or surgically corrected. This is the most common type of permanent hearing loss. Even when speech is loud enough to hear, it may still be unclear or muffled. Mixed hearing loss occurs when conductive and SNHL are both experienced. In other words, there may be damage in the outer or middle ear and in the inner ear (cochlea or auditory nerve). An example would be if an individual who typically has SNHL experienced a middle-ear infection, which could then lead to a mixed hearing loss (Association, 2023). From an etiological perspective, sensorineural hearing loss can be further classified into two major categories: hereditary and acquired. Hereditary deafness results from mutations in pathogenic genes within germ cells and is divided into syndrome-type deafness (associated with systemic organ disorders such as retinal degeneration or vestibular injury) and non-syndrome-type deafness (characterized solely by auditory dysfunction without extrinsic ear symptoms), accounting for more than half of all congenital permanent hearing impairments. Acquired sensorineural hearing loss primarily stems from noise exposure, ototoxic drugs, viral labyrinthitis, or age-related cochlear degeneration (Bayazit and Yilmaz, 2006).

However, for the majority of sensorineural deafness cases in which the pathology resides in the inner ear, auditory nerve, or auditory cortex, particularly those resulting from irreversible damage or loss of inner ear hair cells, existing treatments yield minimal results (Nieman and Oh, 2020) (Figure 1B). Pharmacological interventions are effective only for early reversible hearing loss and cannot repair necrotic inner ear hair cells (Rousset et al., 2022). As an alternative non-surgical option, hearing aids remain challenged in complex noise environments, and non-professional fitting carries risks of exacerbating hearing loss (Jorgensen and Wu, 2023). While cochlear implants could restore hearing, they present issues such as high cost, surgical trauma, and the need for long-term post-operative rehabilitation training (Magro et al., 2018). Furthermore, although gene therapy for hereditary deafness has entered clinical trials, unified clinical standards have yet to be established, and its application is currently limited to specific gene mutation types (Fan et al., 2025).

Vestibular system disorders also impose a significant health burden, with associated vertigo, balance impairment, and fall risks substantially impacting patients’ quality of life and safety (Hall et al., 2022). Benign paroxysmal positional vertigo (BPPV), one of the most common peripheral vestibular disorders, has a core pathological mechanism where otolith particles detach and stimulate semicircular canal hair cells, triggering vertigo. Currently, there remains a lack of curative drugs capable of reversing structural damage to the vestibular organ or restoring otolith adhesion function. Otolith repositioning remains the primary etiological treatment, supplemented by central compensatory strategies such as vestibular rehabilitation training (Wang et al., 2014).

Faced with this challenging reality, the need to deeply analyze the mechanisms of auditory and vestibular system disorders and develop effective treatments is increasingly urgent. However, traditional research models, such as animal models and two-dimensional (2D) cell cultures, struggle to meet the demands of elucidating disease mechanisms and developing therapies. Consequently, the otic vitro models centered on three-dimensional (3D) air-liquid interface (ALI) cultured middle ear epithelial cell models and inner ear organoids are rapidly advancing. The 3D ALI culture of middle ear epithelial cells overcomes the limitations of high cellular heterogeneity and low functional simulation. Functional enhancement is achieved through precise cell selection and optimization of the culture system (Mulay et al., 2016; Espahbodi et al., 2021; Mather et al., 2021). Inner ear organoids could simulate the developmental process of the human inner ear, opening a technical pathway for personalized drug screening for inner ear diseases. They have become a crucial bridge connecting basic research and clinical translation (van der Valk et al., 2023b; Xia et al., 2023b; Wang et al., 2025).

## 2 Limitations of traditional research models

Traditional ear research models primarily include 2D cell culture models, animal models, and human tissue specimens. 2D cell culture typically refers to an *in vitro* cultivation method where cells are seeded onto flat substrates such as culture dishes, forming a monolayer on a 2D plane and undergoing intercellular interactions (Shyam et al., 2023). In such culture systems, cells often progressively lose their inherent physiological phenotypes and functional characteristics, leading to significant discrepancies between research outcomes and *in vivo* physiological reality (Zhang et al., 2025). Gibbs et al. revealed that under 2D culture conditions, the formation of viral replication compartments (VRCs) for murine cytomegalovirus (mCMV) are significantly restricted, with no clear VRCs fusion observed. This fails to accurately reflect the virus’s replication capacity and dynamic processes within the host, potentially underestimating its replication activity and pathogenic potential (Gibbs et al., 2013). Kerschner et al. reported in human middle ear epithelial cell cultures that 2D cultured cells generally fail to form a fully functional epithelial barrier with intact tight junctions. Moreover, these cells exhibit impaired development of functional cilia and are unable to recapitulate the authentic mucin expression profile observed *in vivo* (Kerschner, 2007).

Due to species differences, commonly used mouse models of hearing loss exhibit inconsistencies with the genetic characteristics of hearing-impaired populations. Their reported indicators show weak correlation with human clinical phenotypes (Maraslioglu-Sperber et al., 2024). Shapiro et al. introduced the solute carrier family 26 member 4 (*SLC26A4*) gene mutation protein histidine 723 arginine (p.H723R) into a mouse model. No significant phenotypic abnormalities in hearing or vestibular function were observed in these mice, despite this mutation commonly causing hereditary deafness in humans (Shapiro et al., 2013). Furthermore, mouse hair cells exhibit limited regenerative capacity, whereas human hair cells cannot undergo spontaneous repair after injury (Oshima et al., 2009; Li W. et al., 2024). Although zebrafish are widely used in auditory research, their auditory organ structure fundamentally differs from that of mammals. Zebrafish lack a true cochlear structure and primarily rely on the lateral line system to perceive mechanical stimuli (Brehm et al., 2023). Consequently, they struggle to fully mimic human sound conduction and perception mechanisms. Additionally, their auditory-related gene expression and regulatory networks differ significantly from those in humans, limiting the correspondence between their phenotypes and human deafness pathologies (Giffen et al., 2024). Concurrently, due to sample scarcity and ethical constraints, obtaining human inner ear tissue biopsies is highly challenging, and *postmortem* samples could not reflect the dynamic processes of living organisms (Andresen et al., 2024). Therefore, developing novel *in vitro* ear models holds significant importance for elucidating the mechanisms of ear diseases and advancing clinical therapeutic approaches (Jeong et al., 2026).

Research on *in vitro* ear models is undergoing a profound shift from structural simulation to functional recreation. Central to this transformation are models based on 3D ALI culture of middle ear epithelial cells and inner ear organoids. Both models ensure high matching with the physiological characteristics of *in vivo* ear tissue through precise selection of cell types and optimization of stem cell differentiation pathways.

## 3 3D ALI culture model of middle ear epithelial cells

### 3.1 Design principles

**FIGURE 2:**
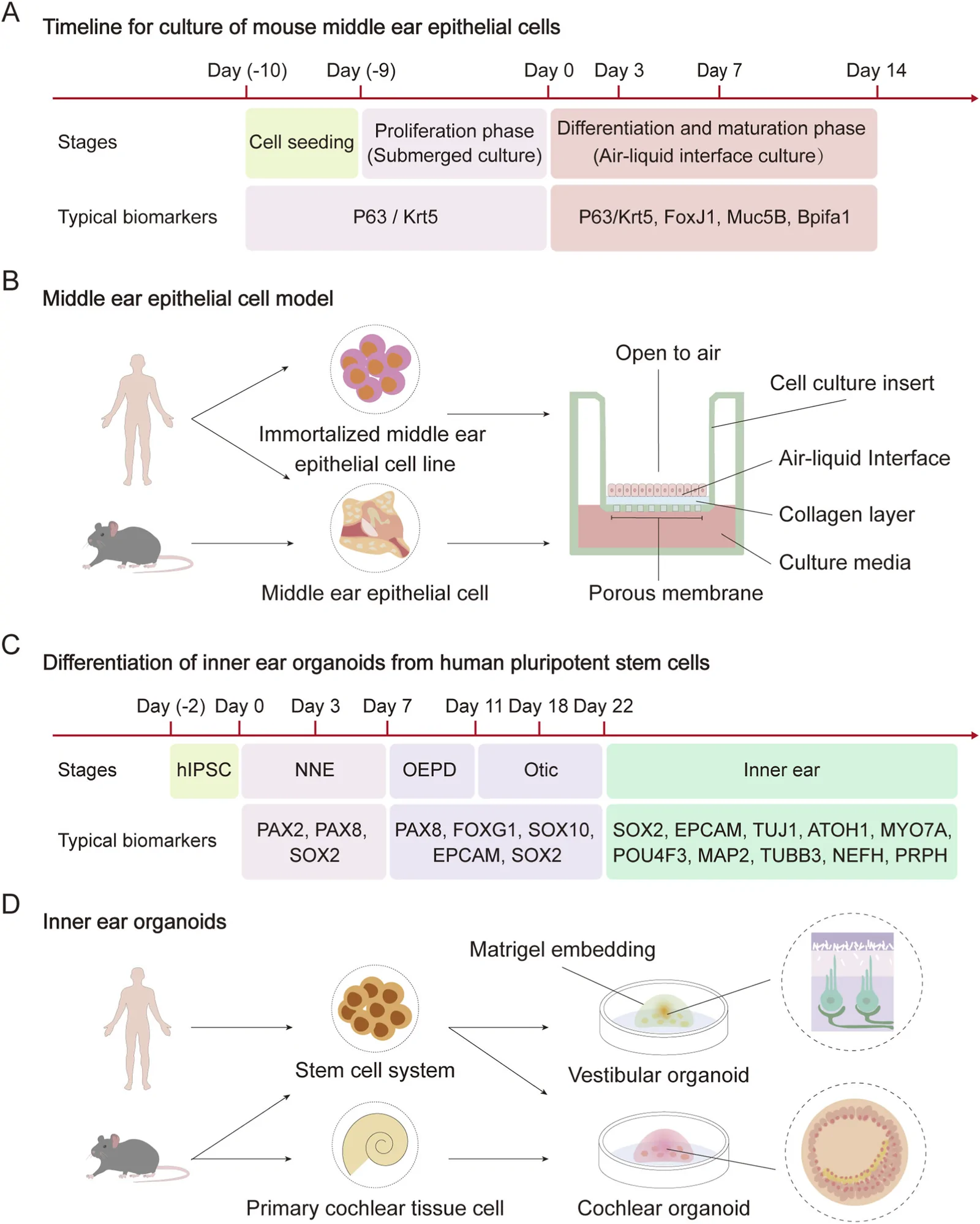
Schematic diagrams of the middle ear epithelial cell model and the inner ear organoid model. **(A)** Timeline of mouse middle ear epithelial cell culture under ALI conditions, showing the proliferation phase (submerged culture) and differentiation/maturation phase (ALI culture), with representative biomarkers. **(B)** ALI culture technology. **(C)** Differentiation roadmap of human pluripotent stem cells toward inner ear organoids, depicting sequential stages from human-induced pluripotent stem cells (hiPSCs) through non-neural ectoderm (NNE), otic-epibranchial placode domain (OEPD), otic vesicle, to mature inner ear structures, with stage-specific biomarkers. **(D)** Organoid culture technology and matrigel embedding method. They are designed to mimic physiological or pathological conditions of the ear for research purposes.

The 3D ALI middle ear epithelial cell model refers to a 3D *in vitro* epithelial cell model cultured under physiologically relevant conditions, such as an ALI, capable of highly simulating the key structures and functions of middle ear epithelium (Chen et al., 2019). The model construction primarily consists of two stages: initial proliferation through immersion culture followed by differentiation and maturation at the Air-Liquid Interface. During this process, biomarkers are expressed in sequence. The proliferative phase is characterized by the expression of P6/Krt5, whereas the differentiation/maturation phase is marked by the expression of FoxJ1, Muc5B, and Bpifa1 (Figures 2A,B). Although ALI culture has been widely used in the construction of various epithelial models, such as those of the respiratory tract and intestine, the middle ear epithelial ALI model exhibits distinct tissue specificity (Baldassi et al., 2021; Sabapaty et al., 2024). Different from the respiratory tract ALI culture centered on the reconstruction of mucociliary clearance function or the intestinal ALI culture oriented towards the polarization of the absorption-secretion barrier, the middle ear epithelial ALI is specifically designed to mimic the physiological characteristics of the middle ear air cavity. The core technical differences are focused on maintaining low mucus secretion, high ion transport activity, and the integrity of the tight junction barrier, so as to reproduce the unique fluid clearance and homeostasis regulation mechanisms of the middle ear (Mulay et al., 2016; Mather et al., 2021).

### 3.2 Fabrication strategies and applications

**FIGURE 3:**
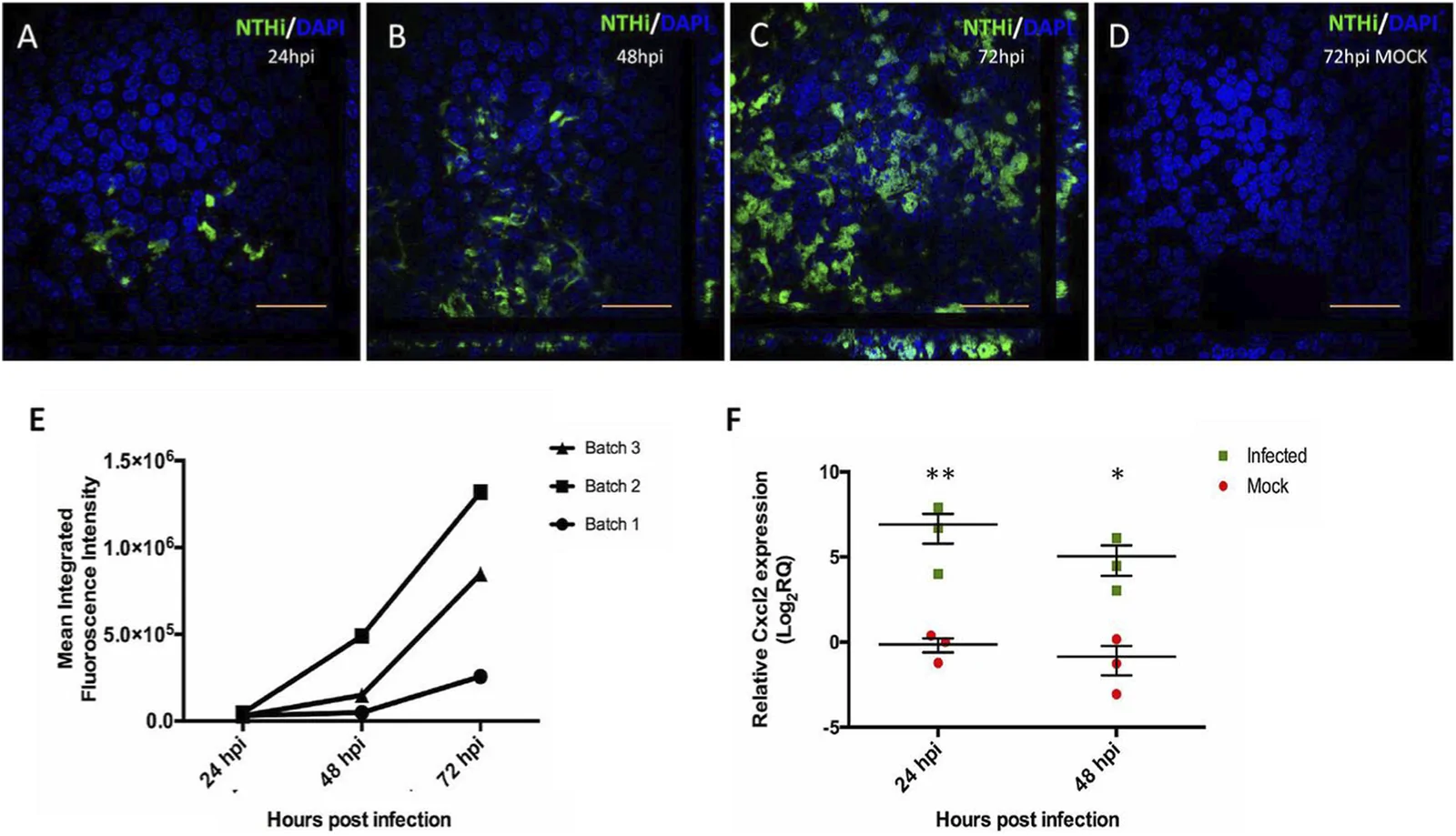
Mouse middle ear epithelial cell (mMEC) cultures serve as an otopathogenic infection model. **(A–C)** High magnification (×60) confocal images representative of three independent batches of ALI day 14 mMECs infected with GFP-tagged NTHi-375SR for 24 h **(A)**, 48 h **(B)** and 72 h **(C)** showing a time-dependent increase in bacterial infection. **(D)** Control ALI day 14 mMECs mock infected for 72 h. Scale bars: 50 μm. **(E)** Increase in mean green fluorescence intensity from 24 hpi to 72 hpi quantified from lower magnification (×10) images from three independent batches, suggesting an increase in the amount of bacteria infecting the cultures with time. **(F)** Relative gene expression of Cxcl2 **(F)** in infected mMEC cultures compared with mock-infected cultures at 24 and 48 hpi. Expression of target gene Cxcl2 was normalised to three endogenous controls; ATP5B, CyC1 and Ppia, and plotted relative to the 24 hpi mock sample. Data was analysed using two tailed Student’s t-test and represented as mean relative quantification (RQ)±s.e.m. For three independent batches of cultures. *P < 0.05, **P < 0.01. This figure was reproduced from (Mulay et al., 2016) under terms of the CC-BY license. https://doi.org/10.1242/dmm.026658. Copyright 2016, The Authors, published by Biologists.

The middle ear epithelial cell model cultured at the 3D ALI is primarily derived from precisely screened primary cells and functionally optimized immortalized cell lines (Baldassi et al., 2021). This model demonstrates promising applications in studying otitis media-related mechanisms, drug screening, and toxicological assessment (Wang et al., 2023) (Figure 3).

Primary cells retain core *in vivo* functions and enable enrichment of functional cell subpopulations via specific markers. Mulay et al. successfully isolated and purified primary cells including basal cells, ciliated cell precursors, and secretory cell precursors from mouse middle ear tympanic membrane mucosa through mechanical dissection combined with marker-based selection. They established an *in vitro* mouse middle ear epithelial cell model (Mulay et al., 2019). Mather et al. developed a novel human middle ear epithelial cell model cultured at the ALI. Experiments demonstrated that this model not only reproduces the complex cellular composition of *in vivo* middle ear epithelium, but also preserves core functions such as mucosal barrier integrity and ciliary motility. Its viability as a functional epithelial barrier was validated through immunohistochemistry, ultrastructural analysis, and membrane conductance measurements (Mather et al., 2021). Furthermore, to address limitations in existing *in vitro* models for otitis media research, Chen et al. employed conditional reprogramming technology. By combining radioactively labeled fibroblast-conditioned medium with Rho kinase inhibitor, they successfully proliferated and differentiated middle ear epithelial cells derived from pediatric cochlear implant surgery patients. After 2–3 weeks of ALI culture, these cells expressed epithelial markers such as Keratin5 (KRT 5), β-tubulin (TUBB), and stably secreted mucins such as Mucin 5B (MUC5B) and Mucin 5AC (MUC5AC). They exhibited distinct apical and basal secretion profiles, with mucins secreted exclusively apically. This provides a more physiologically relevant novel *in vitro* model for otitis media research (Chen et al., 2019).

Immortalized cell lines represent another crucial cellular source for constructing 3D ALI culture models of middle ear epithelial cells. Through genetic modification or optimized culture conditions, immortalized cell lines overcome the passage limitations of primary cells, making them suitable for large-scale experiments. Blaine-Sauer et al. successfully established novel immortalized middle ear epithelial cell lines retaining otitis media-related phenotypes. They proposed a research plan to cultivate these cell lines at the ALI, enabling investigation of inflammatory responses and mucin production under conditions closer to physiological environments. This provides an ideal model for studying inflammatory mechanisms and targeting drug screening (Blaine-Sauer et al., 2023).

## 4 Inner ear organoids

### 4.1 Design principles

Inner ear organoids are defined as 3D miniature organ models generated by inducing pluripotent stem cells, tissue stem cells, progenitor cells, or differentiated cells from healthy or diseased tissues to self-organize and differentiate under *in vitro* 3D culture conditions (Cheng et al., 2026). These structures exhibit organ-level functional mimicry and substantial application potential (Lancaster and Huch, 2019; Zong et al., 2024). Taking the differentiation of human-induced pluripotent stem cells (hiPSCs) into inner ear organoids as an example, this process sequentially involves stages including the non-neural ectoderm (NNE), otic-epibranchial progenitor domain (OEPD), otic vesicle, and inner ear structures. This process is characterized by the temporal expression of stage-specific biomarkers, including PAX2/PAX8/SOX2 in early otic specification, PAX8/FOXG1/SOX10/EPCAM/SOX2 at the otic vesicle stage, and ATOH1/MYO7A/POU4F3, MAP2/TUBB3/NEFH/PRPH in hair cells and neurons during terminal differentiation. Inner ear organoids could be subdivided into vestibular organoids and cochlear organoids (van der Valk et al., 2023a). Currently, inner ear organoid sources primarily focus on stem cell systems and primary cultures derived from cochlear tissue (Figures 2C,D). Based on stem cell type, they could be categorized as pluripotent stem cells (PSCs) derived or adult stem cells (ASCs) derived. PSCs encompass embryonic stem cells (ESCs) and induced pluripotent stem cells (iPSCs) (Jeong et al., 2018). ESCs exhibit strong self-renewal capacity and high multipotent differentiation potential, demonstrating stable differentiation efficiency and practical value in in vitro organoid construction research. iPSCs possess robust proliferative capacity and could undergo directed differentiation, finding extensive applications in constructing specific pathological models and personalized drug screening (Lucassen et al., 2026). Commonly used ASCs primarily include two major categories: tissue-specific progenitor cells and mesenchymal stem cells. Tissue-specific progenitor cells offer advantages of high tissue specificity and shorter differentiation pathways, while mesenchymal stem cells expand research applicability through their multipotent differentiation potential (Table 1).

**TABLE 1:** Sources, methods, characteristics, and applications of middle ear epithelial cell and inner ear organoid models.

Model		Source type	Cell source	Differentiation/Induction method	Culture complexity	Representative cell populations	Applications	Major limitations	References
3D ALI culture of middle ear epithelial cells		Primary cells	Mouse middle ear tympanic membrane mucosal cells	Mechanical dissection and marker-based selection; ALI culture on transwell inserts	Moderate	Basal cells, ciliated cell precursors, secretory cell precursors	Basic research on the function of middle ear epithelial cells	Limited access to primary cells	Mulay et al. (2019)
			Human middle ear epithelial cells	ALI culture; conditional reprogramming	Moderate	Basal cells, ciliated cells, secretory cells	Research on middle ear physiological mechanisms, evaluation of mucosal barrier function	Inability to fully replicate *in vivo* 3D architecture; lack of immune components	Mather et al. (2021); Chen et al. (2019)
		Immortalized cell lines	Human middle ear epithelial cells	Genetic modification; ALI culture	Low	Epithelial cells retaining otitis media-related phenotypes	Research on inflammatory mechanisms of otitis media and high-throughput screening of targeted drugs	Potential loss of primary cell characteristics over passages	Blaine-Sauer et al. (2023)
Inner ear organoids	Vestibular organoids	Pluripotent stem cells (PSCs)	Mouse embryonic stem cells (mESCs)	3D serum-free floating culture; temporal modulation of bone morphogenetic protein (BMP), transforming growth factor-β (TGF-β), and fibroblast growth factor (FGF) pathways	High	Vestibular-like sensory epithelia (type I and type II vestibular hair cells, supporting cells)	Vestibular system developmental mechanism analysis	Species differences; incomplete maturation; predominantly vestibular rather than cochlear fate	Matern and Heller. (2025)
			Human-induced pluripotent stem cells (hiPSCs)	Directed differentiation; 3D culture system; mimicking fetal inner ear developmental microenvironment signals	High	Hair cells, supporting cells, and neurons	Vestibular development; balance disorder modeling; drug screening	Vestibular-like organoids rarely acquire cochlear fate spontaneously; significant inter-batch heterogeneity; lack of vascularization	Mattei et al. (2019); van der Valk et al. (2025)
	Cochlear organoids	PSCs	mESCs	3D directed differentiation; modified signaling pathway protocols to promote cochlear-like hair cell differentiation	High	Cochlear-like hair cells, supporting cells, spiral ganglion neuron-like cells	Cochlear development; hereditary deafness modeling	Low efficiency in acquiring cochlear fate; slow hair cell maturation	Koehler et al. (2013); Koehler and Hashino. (2014)
			hiPSCs	Small molecule optimization	High	Hair cells	Personalized pathology model construction; hereditary deafness modeling	Low differentiation efficiency; prolonged culture duration	Moore et al. (2023)
		Adult stem cells (ASCs)	Lgr5+ cochlear progenitor cells in mice	3D matrigel culture; Wnt/YAP pathway activation; staged 3D co-culture	Moderate	Hair cells with intact stereociliary bundles and mechanotransduction function	Research on inner ear targeted differentiation; elucidation of mechanisms underlying sensory cell regeneration; models of synaptic function-related diseases	Species differences; limited scalability	Carpena et al. (2025); Xia et al. (2023a)
			Human bone marrow-derived mesenchymal stem cells	*In vitro* directed trans-lineage induction	Moderate	Inner ear sensory cell-like cells (including cochlear hair cell-like cells)	Research on inner ear damage repair and exploration of alternative cell sources	Unstable differentiation efficiency; functional maturation pending validation	Duran Alonso et al. (2012)
		Primary cochlear tissue cells	Mouse cochlear auditory sensory epithelial progenitor cells	Hydrogel encapsulation; *in vitro* proliferation and differentiation induction	Moderate	Embryonic cochlear tissue-like cellular architecture	Research on the tissue-specific functions of the inner ear	Difficulties in primary cell acquisition; enzymatic dissociation may disrupt cell-cell junctions	Hu et al. (2024)
			Mouse cochlear basilar membrane stem cells	Spontaneous cell aggregation; matrix metalloproteinase 9 (MMP-9)-mediated extracellular matrix remodeling	Moderate	Cochlear organoids containing functionally mature hair cells	Research on the mechanism of hair cell regeneration in the cochlea	Significant batch-to-batch variability in self-assembly efficiency	Li et al. (2025a)

### 4.2 Fabrication strategies and applications of vestibular organoids

Vestibular organoids have emerged as a core tool for studying vestibular system developmental mechanisms, modeling balance disorder-related diseases, and conducting drug screening, with their construction primarily focused on stem cell systems.

For investigations into developmental mechanisms, Mikosz et al. first established a 3D serum-free floating culture system using the embryoid body-like aggregates with quick reaggregation (SFEBq) method. They subsequently performed precise modulation of core developmental signaling pathways, including the bone morphogenetic protein (BMP), transforming growth factor-β (TGF-β), and fibroblast growth factor (FGF) pathways. With this optimized *in vitro* system and stepwise signaling control, they successfully generated inner ear vestibular organoids from mouse embryonic stem cells (mESCs) (Koehler et al., 2013). Matern et al. employed single-cell transcriptomics to first map the developmental trajectory of mESCs-derived vestibular-like inner ear organoids, revealing the differentiation pathway from cochlear precursor cells to functional cells. They further discovered synchronous generation of cochlear precursors and neural crest cells. This finding first elucidates the molecular mechanism of neurosensory co-development in organoids, exhibiting high similarity to embryonic neural crest migration patterns, and provides a novel model for studying inner ear innervation (Matern and Heller, 2025).

**FIGURE 4:**
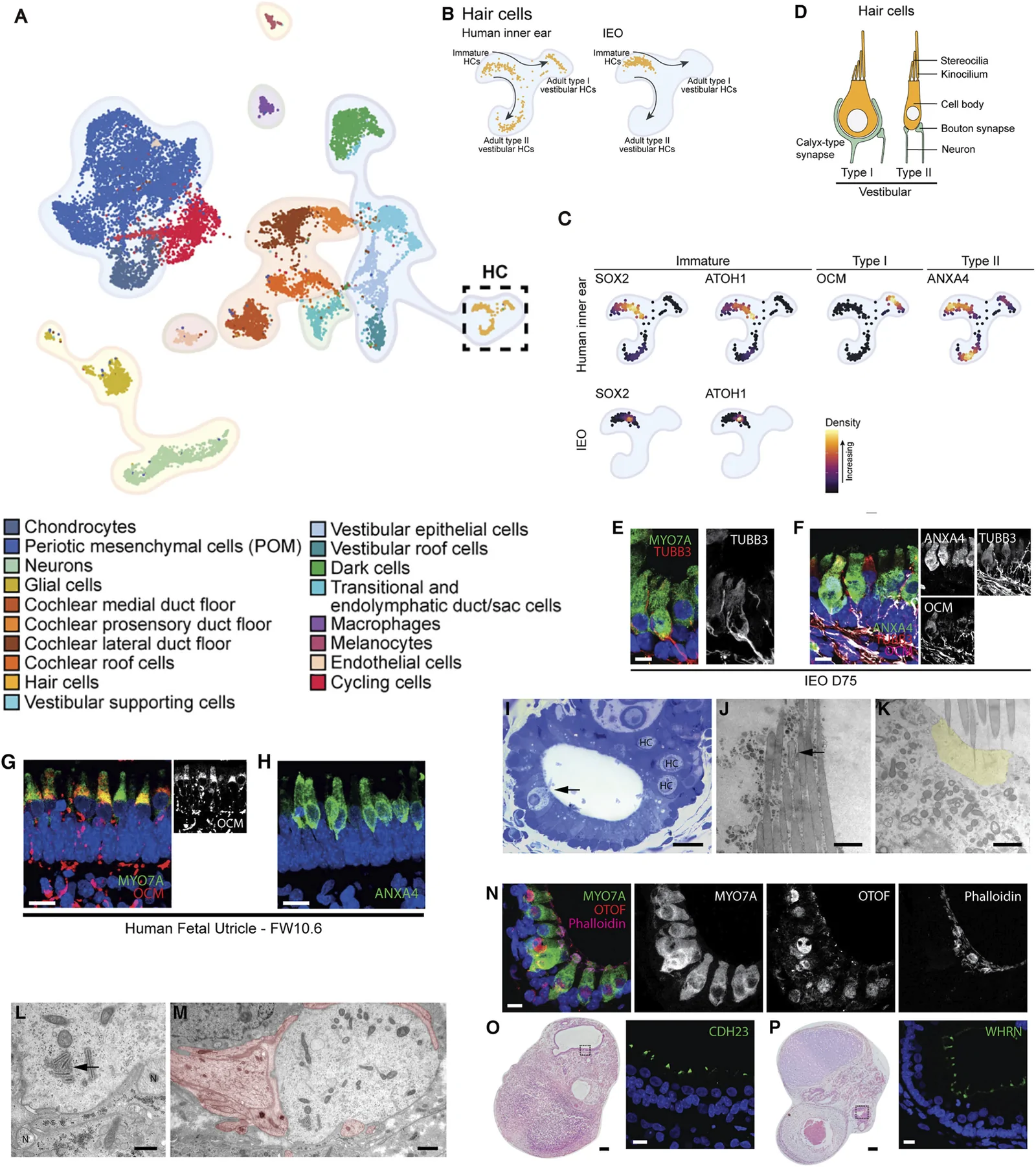
Immature type I and type II vestibular hair cells in Inner Ear Organoids (IEOs). **(A)** UMAP plot of annotated human inner ear atlas highlighting hair cells (HCs). **(B)** UMAP plot comparing reference hair cell population (left) and mapped IEO-derived hair cell population (right) using Symphony. **(C)** Density plots of reference hair cell population (left) for immature, type I, and type II vestibular markers, and density plots of mapped IEO-derived hair cells showing expression of immature markers. **(D)** Schematic depicting morphological differences between type I and type II vestibular hair cells. **(E)** Calyx-type synapse (TUBB3^+^) surrounding a hair cell (MYO7A^+^). Scale bar, 10 μm. **(F)** Expression of ANXA4 and OCM in hair cells of a D75 IEO, showing subsets with none, either, or both. Scale bar, 10 μm. **(G)** OCM expression in hair cells of human fetal utricle (FW10.6). Scale bar, 10 μm. **(H)** ANXA4 expression in human fetal utricle (FW10.6). Scale bar, 10 μm. **(I)** Methylene blue-azure II staining of a D74 IEO-vesicle containing hair cells (arrow and “HC”). Scale bar, 20 μm. **(J)** Hair bundle with stereocilia and kinocilium cross-section (arrow). Scale bar, 1 μm. **(K)** Apical region of a hair cell with cuticular plate (yellow) and stereociliary rootlets. Scale bar, 1 μm. **(L)** Basal region of a hair cell showing synaptic ribbons (arrow) and bouton-like synapses (“N”). Scale bar, 500 nm. **(M)** Transection of type I vestibular hair cell almost entirely surrounded by calyx-type synapse (in red). Scale bar, 500 nm. **(N)** OTOF expression in MYO7A^+^ hair cells. Scale bar, 10 μm. **(O)** H&E overview with CDH23 in stereocilia by IHC. Scale bars: H&E, 100 μm; IHC, 20 μm. **(P)** H&E overview with WHRN in stereocilia by IHC. Scale bars: H&E, 100 μm; IHC, 20 μm. H&E and IHC images are representative of n ≥ 6 aggregates (≥2 experiments) (E, F, and N–P) or n ≥ 2 human inner ear tissues of matching fetal age **(G, H)**. TEM images are representative of n ≥ 3. Reproduced under terms of the CC-BY license (van der Valk et al., 2023b), with layout modifications made to improve compactness and readability. DOI: https://doi.org/10.1016/j.celrep.2023.112623. Copyright 2023, The Authors, published by Cell Press.

In disease modeling and drug screening, Mattei et al. utilized a rotary cell culture system (RCCS) to induce inner ear vestibular organoids from human PSCs. The vestibular hair cells within these organoids exhibit high morphological and physiological similarity to human fetal vestibular hair cells and form otolith-like structures. This way serves as an indispensable tool for deciphering the developmental mechanisms of the human vestibular system. Beyond fundamental developmental research, it establishes a robust experimental foundation for modeling the pathological processes of balance disorders and their associated genetic conditions, most notably Usher Syndrome. Furthermore, this platform enables reliable high-throughput drug screening to identify novel therapeutic candidates for these debilitating disorders (Mattei et al., 2019). By mimicking embryonic developmental signals, Van et al. induced the generation of vestibular organoids in a 3D culture system. These organoids, comprising hair cells, supporting cells, and neurons, reproduce the core features of the human vestibular organ. This approach overcomes the species-specific limitations of animal models, providing an innovative platform for research and therapeutic translation related to balance disorders (van der Valk et al., 2025) (Figure 4).

### 4.3 Fabrication strategies and applications of cochlear organoids

Cochlear organoids are derived through two primary pathways: stem cell systems and primary cultures from cochlear tissues. Each approach offers distinct technical advantages and application scenarios.

Koehler et al. employed mESCs in 3D culture with directed differentiation, successfully generating cochlear organoids containing inner ear-specific cell subtypes such as hair cells and supporting cells. These organoids mimic the structural and developmental characteristics of the *in vivo* cochlea (Koehler and Hashino, 2014). Moore et al. successfully constructed high-fidelity cochlear organoids from human iPSCs, which contained hair cells, supporting cells, and innervating neurites. Notably, the differentiated hair cells exhibited morphology, molecular marker expression, and functional characteristics similar to *in vivo* cochlear hair cells, including electrophysiological properties consistent with both inner and outer hair cells (Moore et al., 2023). Carpena et al. successfully constructed cochlear organoids with complete stereociliary bundles and mechanotransduction function using mouse Lgr5^+^ cochlear progenitor cells (inner ear-derived ASCs), validating the targeted differentiation potential of tissue-specific progenitor cells (Carpena et al., 2025). Xia et al. isolated Lgr5^+^ cochlear progenitor cells from mouse inner ears. By optimizing culture media to activate the Wnt/YAP signaling pathway and enhance proliferation, followed by induction via a staged 3D co-culture system, they generated cochlear organoids reproducing functional synaptic connections of the peripheral auditory circuit (Xia et al., 2023a). Duran Alonso et al. successfully obtained inner ear sensory cell-like cells (including cochlear hair cell-like cells) by *in vitro* induction of human bone marrow-derived mesenchymal stem cells. This study clarified the regulatory relationship between key induction factors and differentiation stages during trans-lineage differentiation. Its paracrine effects and multi-directional differentiation characteristics provide an alternative model for inner ear injury repair research (Duran Alonso et al., 2012).

Compared to directed differentiation from PSCs, cochlear organoids constructed from primary cells derived from cochlear tissue possess unique advantages. Although obtaining primary cells requires enzymatic dissociation, potentially leading to partial loss of original intercellular junctions, they retain the developmentally specific imprint of inner ear tissue. Under appropriate 3D culture conditions, these cells could efficiently self-assemble into organoids that closely resemble the *in vivo* cochlea in both structure and cellular composition, based on their intrinsic developmental program. Hu et al. isolated inner ear progenitor cells from the auditory sensory epithelium of murine cochleae. These progenitor cells were encapsulated in hydrogels, and subsequent *in vitro* culture induced their proliferation and differentiation, leading to the formation of cochlear organoids. Notably, the cellular architecture of these organoids exhibits a high degree of similarity to that of embryonic cochlear tissues (Hu et al., 2024). Li et al. isolated cochlear stem cells from the basilar membrane of wild-type mouse cochleae. Through spontaneous cell aggregation and extracellular matrix remodeling mediated by matrix metalloproteinase 9 (MMP-9), these cells self-organized and ultimately differentiated into cochlear organoids containing functionally mature hair cells. Collectively, these studies demonstrate that leveraging the intrinsic developmental programs of primary cells for self-organization, rather than relying solely on directed differentiation pathways from pluripotent stem cells, enables the construction of cochlear organoids with higher simulation fidelity (Li N. et al., 2025).

Currently, cochlear organoid models have become powerful tools for fundamental and disease research in otology. Li et al. utilized cochlear organoid models for auditory regeneration studies, discovering that short-term application of Jagged1-Fc peptide to activate the Jagged1/Notch signaling pathway could indirectly enhance the ability to generate hair cells *in vitro* and *in vivo* by inducing supporting cell proliferation (Li X. J. et al., 2025). In disease modeling, Nie et al. utilized human cochlear organoids to model the coloboma, heart defects, atresia of choanae, retarded growth and development, genital hypoplasia, ear anomalies (CHARGE) syndrome, which arises from mutations in the chromodomain helicase DNA-binding protein 7 (CHD7) gene. This approach revealed the key gene regulatory networks underlying the loss of hair cells and supporting cells induced by these mutations (Nie et al., 2022). Similarly, Tang et al. combined cochlear organoids with single-cell sequencing to elucidate the molecular mechanism by which transmembrane protease serine 3 (TMPRSS3) mutations cause hair cell degeneration, offering new insights into understanding hereditary deafness (Tang et al., 2019).

Regarding clinical applications, cochlear organoids demonstrate significant value in auditory damage repair, personalized medicine, and gene therapy research. In auditory damage repair studies, Sun et al. successfully developed a novel technique to differentiate human PSCs into inner ear neurons. Following cryopreservation and thawing, this method efficiently and reproducibly generates cochlear organoids, providing a new tool for studying and treating sensorineural hearing loss (Sun et al., 2025). For personalized medicine, researchers could construct highly customized disease models *in vitro* by differentiating iPSCs harboring patient-specific mutations into cochlear organoids. Zafeer et al. observed significant developmental abnormalities in cochlear organoids using this technique, directly demonstrating the causal relationship between specific genetic variants and cochlear malformations. This provides an efficient functional validation platform for the precise diagnosis of such hereditary deafness (Zafeer et al., 2025). Based on such models, cochlear organoids could also simulate patient-specific cochlear responses for personalized drug screening and toxicological assessment, accelerating new drug development and clinical translation (Qi et al., 2024). In gene therapy research, cochlear organoids are assuming an increasingly vital role. Although significant progress has been made in adeno-associated virus (AAV) mediated inner ear gene therapy using animal models, challenges persist in clinical translation, with host immune responses to AAV vectors being a key issue. Human cochlear organoids offer an *in vitro* model that circumvents species differences and more closely mimics human physiological conditions. This provides a unique platform for investigating human-specific immune mechanisms triggered by AAV, which is crucial for ensuring the safety and efficacy of future gene therapies (Ishibashi et al., 2023).

## 5 Challenges and future prospects

With continuous advancements in auditory science and regenerative medicine, the development and application of 3D ALI cultured middle ear epithelial cell models and inner ear organoid models have become a key focus of otology research. These models present new opportunities for elucidating the mechanisms of otologic diseases and innovating therapeutic strategies, while also posing challenges. The former offers advantages of simplicity, reproducibility, and lower cost, making it an important tool for early drug screening and toxicity assessment. However, it faces limitations in simulating the 3D structure of the middle ear and complex intercellular interactions. In contrast, inner ear organoids offer superior advantages in cellular diversity, tissue architecture, and physiological function, better mimicking the *in vivo* microenvironment and signaling pathways. However, they are hampered by prolonged culture periods, demanding technical procedures, and substantial batch-to-batch variation. These limitations arise primarily from the complex self-organization process, slow differentiation and maturation kinetics, and high sensitivity to *in vitro* culture conditions, which restrict their large-scale and standardized application (Wang et al., 2023).

### 5.1 Comparative analysis of ALI-cultured middle ear epithelial models and inner ear organoids

ALI culture of middle ear epithelial cell model exhibits significant differences from inner ear organoids in terms of cellular composition, physiological relevance, reproducibility, disease modeling capabilities, and translational application value (Table 2). The ALI culture of middle ear epithelial cell model highly accurately replicates mucosal barrier function and ciliary activity with excellent reproducibility, making it a high-throughput platform for otitis media and conductive hearing loss research. However, it is not suitable for studies on sensorineural or vestibular disorders. In contrast, inner ear organoids exhibit superior physiological fidelity in modeling hereditary deafness, vestibular dysfunction, and hair cell regeneration. Inner ear organoids derived from hiPSCs enable personalized phenotypic analysis and verification of genetic variations. However, the widespread application of inner ear organoids is constrained by multiple interrelated challenges, which will be systematically discussed in the next section.

**TABLE 2:** Comparative analysis of ALI-cultured middle ear epithelial models and inner ear organoids.

Parameter		ALI-cultured middle ear epithelial model	Inner ear organoids	References
Cellular composition		Epithelial cells (basal cells, ciliated cells, secretory cells)	Hair cells, supporting cells, neurons	Mulay et al. (2019); van der Valk et al. (2025)
Physiological relevance		Moderate (mucosal barrier, ciliary function)	High (sensory transduction, neuronal connectivity)	Mather et al. (2021); Moore et al. (2023)
Culture complexity		Low to moderate	High	Mather et al. (2021); Koehler and Hashino. (2014)
Reproducibility		Excellent; minor batch variation, stable passaging for primary/immortalized cells	Poor; severe inter-batch heterogeneity from stem cell and matrix batch differences	Mather et al. (2021); Hocevar et al. (2021)
Disease-modeling capability		Conductive hearing loss, otitis media, mucosal barrier injury; cannot model sensorineural/vestibular disorders	Hereditary sensorineural deafness, vestibular dysfunction, hair cell damage; not for middle ear inflammation	Chen et al. (2019); Nie et al. (2022)
Translational applications	Drug screening	High-throughput, low-cost platform for anti-otitis media and mucosal toxicity screening	High cost and unstable differentiation; only small-scale verification of inner ear otoprotective compounds	Blaine-Sauer et al. (2023); Qi et al. (2024)
	Gene therapy evaluation	Rarely used; only for gene intervention targeting middle ear epithelium inflammation	Applied to basic research of hereditary deafness gene mechanism and *in vitro* gene therapy verification	Ishibashi et al. (2023)
	Personalized medicine	Limited utility; only for individual middle ear mucosal inflammation analysis	Patient human-induced pluripotent stem cells (hiPSCs)-derived organoids recapitulate unique patient pathogenic phenotypes	Chen et al. (2019); Zafeer et al. (2025)

### 5.2 Current challenges and limitations of inner ear organoids

The challenges discussed below are not merely technical hurdles but fundamental constraints that reflect the inherent difficulty of recapitulating the complex spatiotemporal dynamics of inner ear development *in vitro*. Addressing these limitations will require not only incremental optimization of existing protocols but also paradigm shifts in how we approach organoid engineering.

#### 5.2.1 Incomplete cellular composition and insufficient morphological reduction

Although inner ear organoids can differentiate into various types of inner ear cells, their cellular composition exhibits fundamental differences from that of the native inner ear in terms of relative proportions and diversity (Rong et al., 2026). Inner ear organoids consist of sensory and non-sensory epithelial cells, otic neuroblasts, neurons, glial cells, mesenchymal tissue, as well as non-targeted tissues (such as cartilage and epidermis) that develop collectively *in vitro* (Pianigiani and Roccio, 2024). Single-cell transcriptome analysis further revealed that, in addition to targeting peripheral mesenchymal cells associated with the inner ear, non-targeted cell types such as skull skeletal muscle cells, endothelial cells, and ependymal cells were also detected within the inner ear organoids (van der Valk et al., 2023a). More importantly, existing differentiation protocols produce potential “general-purpose” inner ear cells that lack full specialization into vestibular or cochlear cells. Furthermore, the organoids lack vascular endothelial cells, pericytes, and immune cell populations. These cells are essential for nutrient exchange, homeostasis, and immune regulation (Takebe and Wells, 2019; Teng et al., 2021; Zhao et al., 2022; Rong et al., 2026).

Unlike the precise cellular arrangement and stratified structure of the cochlear spiral organ *in vivo*, the spatial distribution of cells in inner ear organoids exhibits significant randomness and cannot replicate the ordered structures of the cochlea, such as the lamellar bands and vascular patterns. The fundamental reason for these morphological defects lies in the inability of the *in vitro* self-assembly process to accurately mimic the complex spatiotemporal developmental signals and physicochemical microenvironment present *in vivo* (Moore et al., 2023; Pianigiani and Roccio, 2024).

#### 5.2.2 Low differentiation efficiency and prolonged culture cycle

Current experimental protocols for preparing inner ear organoids still require cumbersome operational procedures, typically involving multiple steps such as cell expansion and directed differentiation. These approaches are not only time-consuming and labor-intensive but also inefficient, with limited capacity to obtain sufficient quantities of fully differentiated hair cells and diverse supporting cell types (Qin et al., 2025). The formation of functional sensory hair cells requires approximately 40 days, and the culture system can maintain them for up to 150 days to support further development (van der Valk et al., 2025). It is noteworthy that the maturation of hair cells in cochlear organoids proceeds extremely slowly, typically requiring up to 200 days to express the mature outer hair cell marker Prestin (Saeki et al., 2026). This prolonged cycle not only increases experimental costs and contamination risks, but also complicates research on hearing impairments and long-term pharmacological effects.

#### 5.2.3 Cell heterogeneity and inter-batch variability

Inner ear organoids produced using standard protocols exhibit significant heterogeneity in terms of size, morphology, and efficiency. In particular, when the formulations of commercial reagents change, the organoid culture paradigm exhibits high heterogeneity at every stage; under identical culture conditions, organoid yield can range from 0% to over 90% (Koehler et al., 2017; Rossi et al., 2018; Roccio and Edge, 2019; Pianigiani and Roccio, 2024). In addition to the target cell types found in cochlear and vestibular organoids (hair cells, supporting cells, and neurons), organoids often contain off-target tissues such as cartilage and epidermis. Batch-to-batch variations in extracellular matrix components, such as Matrigel, further exacerbate this problem (Hocevar et al., 2021).

#### 5.2.4 Incomplete maturation and functional defects

Although inner ear organoids replicate many aspects of inner ear development *in vivo*, they consistently exhibit characteristics of incomplete maturation (Cao et al., 2026). Although inner ear organoids express early hair cell markers such as MYO7A and SOX2, they often fail to develop fully mature cilia bundles with normal mechanoelectrical transduction functions. Under resting conditions of supporting cells, the hair cells in inner ear organoids remain immature. Transcriptomic analysis demonstrates that the sensory epithelium derived from inner ear organoids maintains an immature transcriptional state even during late culture stages (Matern and Heller, 2025).

#### 5.2.5 Absence of vascularization and defects in the blood-labyrinth barrier

Vascularization remains a major technical challenge in inner ear organoid research. Most tissues thicker than 400 µm require a functional vascular system to ensure an adequate supply of nutrients and oxygen, but current inner ear organoids lack this critical component (Quintard et al., 2024). The blood-labyrinth barrier is a specialized structure composed of endothelial cells, pericytes, and perivascular resident macrophage-like melanocytes. It plays a critical role in maintaining inner ear homeostasis and understanding drug delivery mechanisms, as well as the pathogenesis of ototoxicity and inner ear disorders (Cosentino et al., 2024). The absence of vascularization limits the size of organoids, restricts nutrient diffusion, and impedes research on the blood-labyrinth barrier (Rong et al., 2026).

#### 5.2.6 Insufficient repeatability and scalability

The insufficient reproducibility and scalability in inner ear organoid production pose significant barriers to both basic research and clinical translation. The non-reproducibility of differentiation protocols and the lack of standardization represent the core challenges currently faced. There is currently no internationally recognized quality evaluation standard for inner ear organoids, which complicates result comparisons across laboratories and the reproducible validation of findings (Pianigiani and Roccio, 2024; Qi et al., 2024). Recent research has primarily focused on developing more refined culture systems, including the use of synthetic hydrogels as alternatives to Matrigel, as well as the application of 3D bioprinting and microfluidic technologies to achieve standardized and scalable organoid production (Shen et al., 2025; Chen et al., 2026).

#### 5.2.7 Insufficient integration with the natural tissue environment

Current inner ear organoids struggle to fully replicate the multi-layered composite microenvironment of the natural inner ear. Existing culture systems can only generate structures resembling the vestibular apparatus and immature cochlear hair cells, lacking the ability to simulate *in vitro* vascular networks. Furthermore, they fail to reconstruct the orderly cellular arrangement and complete intercellular synaptic connections of the Corti apparatus, as well as the complex extracellular matrix components and associated vascular architecture required *in vivo*. Additionally, static three-dimensional cultures cannot mimic critical biophysical signals unique to the natural cochlea, such as fluid dynamics and dynamic mechanical stimuli, nor accurately replicate the specialized ion-rich microenvironment of the endolymph. These limitations significantly hinder the functional maturation of hair cells. In summary, incomplete cell differentiation and the inability to recreate the native physiological microenvironment *in vitro* remain fundamental bottlenecks that must be overcome in the development of inner ear organoid models (Cao et al., 2026).

### 5.3 Future perspectives for *in vitro* ear models

In recent years, biomedical research paradigms have shifted toward reducing animal experimentation and developing more efficient, standardized *in vitro* models. Against this backdrop, countries like the United States and the United Kingdom have introduced significant policy and funding initiatives. For instance, the U.S. National Institutes of Health (NIH) invested $87 million to establish the “National Center for Standardized Organoid Models,” aiming to advance the standardization and scaled application of organoid technology (Health.N.I.o, 2025). Concurrently, the United Kingdom announced a £75 million funding initiative, outlining a strategic roadmap and timeline for replacing, reducing, and refining animal testing in biomedical research (Department for Science, 2025). Collectively, these initiatives provide robust institutional and resource frameworks to advance emerging alternative technologies like organoids.

**FIGURE 5:**
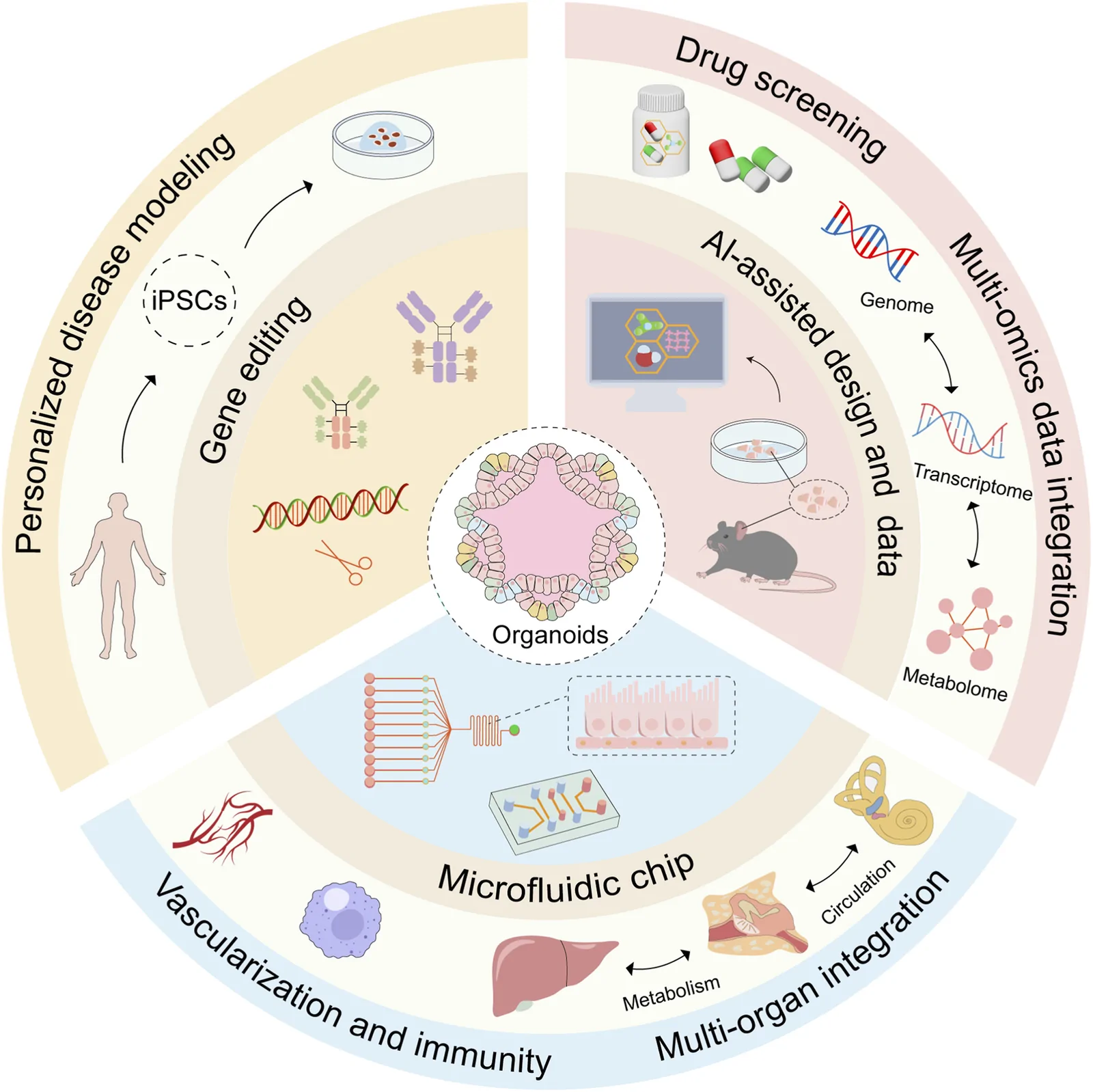
The Frontier directions of ear external model development. The diagram systematically depicts the multi-dimensional applications and technological innovations of organoids. (1) Personalized disease modeling, where patient-specific organoid models are engineered from iPSCs combined with gene editing tools to dissect disease mechanisms at the cellular and molecular levels; (2) Vascularization, immunity, and multi-organ integration research, which leverages microfluidic chips and platforms to recapitulate physiological microenvironments, facilitate organoid-immune cell co-culture, and mimic inter-organ metabolic crosstalk and circulatory interactions. (3) AI-driven drug screening, empowered by artificial intelligence-assisted experimental design and multi-omics data integration (genomics, transcriptomics, and metabolomics) for in-depth mechanistic analysis of drug efficacy and toxicity. This integrated framework highlights the transformative potential of organoid technology in bridging basic research and clinical translation.

Future development of *in vitro* ear models will focus on precise microenvironment simulation, integration of gene editing, and AI-assisted design (Figure 5). Cochlear organoid-on-a-chip technology based on microfluidic chips holds promise as a powerful tool for achieving vascularized dynamic simulation of the cochlear microenvironment and studying multi-tissue interactions (Rong et al., 2026). This technology employs microfluidic systems to precisely regulate cell culture environments, aiming to mimic complex intercellular signaling pathways. This facilitates functional reconstruction of cochlear tissues and enables real-time monitoring of their dynamic responses (Deng et al., 2023; Hu et al., 2024; Casanova et al., 2026; Choudhery et al., 2026). Precise editing of pluripotent stem cells using gene editing technologies like clustered regularly interspaced short palindromic repeats CRISPR-associated protein 9 (CRISPR/Cas9) enables the construction of ear organoids harboring specific pathogenic gene mutations. This provides a novel platform for studying the mechanisms and simulating the phenotypes of diseases such as hereditary deafness (Tang et al., 2019; Li S. et al., 2024). This approach not only aids in elucidating gene functions and disease mechanisms but also lays the groundwork for evaluating personalized gene therapy strategies (Ueda et al., 2023; Zafeer et al., 2025). Artificial Intelligence (AI)-assisted model design and data analysis represent another significant development direction. Through big data mining and machine learning algorithms, AI technology efficiently parses multi-omics data generated by models, optimizing construction workflows and functional evaluation systems. Furthermore, AI demonstrates promising applications in drug screening, novel target discovery, and predicting model dynamics, offering new avenues for precision diagnostics and therapeutics in otologic diseases (Demchenko et al., 2025; Diosdi et al., 2025; Iftode et al., 2025). For instance, AI-assisted image recognition systems have significantly enhanced the efficiency and accuracy of detecting primary inner ear cells and organoid models (Li Y. T. et al., 2025).

## 6 Conclusion

The 3D ALI culture of middle ear epithelial cell model and inner ear organoid model have overcome the limitations of traditional research models, emerging as core *in vitro* tools bridging fundamental otological research, disease mechanism analysis, and clinical translation. The 3D ALI culture of middle ear epithelial cell model, with its advantages of ease of operation, high reproducibility, and relatively low cost, has become a standardized platform for otitis media pathology research, early drug screening, and toxicity assessment. Inner ear organoids, characterized by their high fidelity in mimicking *in vivo* cellular composition, tissue architecture, and physiological functions, have achieved key breakthroughs in areas such as cochlear and vestibular developmental mechanisms, modeling of hereditary deafness, hair cell regeneration, and safety evaluation of gene therapies (Zhi et al., 2026). They provide a more human-relevant experimental system for research on sensorineural hearing loss and vestibular disorders.

The field is currently transitioning from modeling single structures to functional reconstruction and precise microenvironment reconstruction, with research priorities shifting from exploring fundamental mechanisms to personalized medicine and clinical applications. However, the development of *in vitro* ear models still faces multidimensional challenges. At the model-building level, existing systems still have significant shortcomings in terms of structural complexity, vascularization, innervation, and simulation of the immune microenvironment. Although inner ear organoids can reproduce some developmental features, their cellular composition remains incomplete, and their morphological fidelity is insufficient. Their compact aggregate structure also limits effective drug penetration and in-depth detection. At the organoid level, culture cycles are long, and differentiation efficiency is low. Differentiation outcomes vary greatly among different cell lines, making efficient and stable directed differentiation a major technical challenge. At the research paradigm level, there is a lack of a unified system for functional maintenance and assessment during long-term culture. Different laboratories employ varying stem cell lines, differentiation protocols, culture medium formulations, and analytical methods, resulting in a lack of standardized endpoints for evaluating organoid quality. These issues limit the comparability of research results and the reliable evaluation of drug efficacy. These challenges are interrelated and mutually reinforcing. Their root cause lies in the difficulty of precisely replicating the highly ordered developmental signals and complex physicochemical microenvironment found *in vivo* during the *in vitro* self-assembly process. Systemic breakthroughs through interdisciplinary collaboration and technological innovation are urgently needed.

In the future, this field will focus on three core trends: precise microenvironment simulation, accurate gene editing modeling, and AI-assisted optimization. Leveraging cross-disciplinary technologies such as organoid chips, CRISPR precision editing, multi-omics, and AI data analysis, it will drive the advancement of *in vitro* models toward multifunctional integrated systems featuring vascularization, innervation, immune co-culture, and multi-organ integration. As the technological framework matures and multi-model synergistic applications expand, *in vitro* models of the middle and inner ear will deepen our understanding of otologic disease pathologies (Saeki et al., 2026). This will accelerate targeted drug development, validate gene therapies, and advance personalized treatment protocols. Ultimately, these advancements will deliver safer, more efficient, and precise therapeutic strategies for patients with hearing loss and vestibular disorders, significantly improving their quality of life and clinical outcomes.
